# Genetic Structure in the Northern Range Margins of Common Ash, *Fraxinus excelsior* L.

**DOI:** 10.1371/journal.pone.0167104

**Published:** 2016-12-01

**Authors:** Mari Mette Tollefsrud, Tor Myking, Jørn Henrik Sønstebø, Vaidotas Lygis, Ari Mikko Hietala, Myriam Heuertz

**Affiliations:** 1 Norwegian Institute of Bioeconomy Research, Ås, Norway; 2 Institute of Botany of Nature Research Centre, Vilnius, Lithuania; 3 Department of Forest Ecology and Genetics, INIA-Forest Research Centre, Madrid, Spain; 4 BIOGECO, INRA, Univ. Bordeaux, Cestas, France; Technical University in Zvolen, SLOVAKIA

## Abstract

During post glacial colonization, loss of genetic diversity due to leading edge effects may be attenuated in forest trees because of their prolonged juvenile phase, allowing many migrants to reach the colonizing front before populations become reproductive. The northern range margins of temperate tree taxa in Europe are particularly suitable to study the genetic processes that follow colonization because they have been little affected by northern refugia. Here we examined how post glacial range dynamics have shaped the genetic structure of common ash (*Fraxinus excelsior* L.) in its northern range compared to its central range in Europe. We used four chloroplast and six nuclear microsatellites to screen 42 populations (1099 trees), half of which corresponded to newly sampled populations in the northern range and half of which represented reference populations from the central range obtained from previously studies. We found that northern range populations of common ash have the same chloroplast haplotypes as south-eastern European populations, suggesting that colonization of the northern range took place along a single migration route, a result confirmed by the structure at the nuclear microsatellites. Along this route, diversity strongly decreased only in the northern range, concomitantly with increasing population differentiation and complex population substructures, a pattern consistent with a leading edge colonization model. Our study highlights that while diversity is maintained in the central range of common ash due to broad colonizing fronts and high levels of gene flow, it profoundly decreases in the northern range, where colonization was unidirectional and probably involved repeated founder events and population fluctuations. Currently, common ash is threatened by ash dieback, and our results on northern populations will be valuable for developing gene conservation strategies.

## Introduction

The present-day distribution of temperate and boreal tree taxa is largely a result of multiple range changes driven by the Quaternary glaciations [1]. In Europe during the Last Glacial Maximum, temperate tree species were mainly restricted to the southern European peninsulas [2], whereas boreal tree taxa were more widely distributed with patchy occurrences reaching north of the Alps [3, 4]. At the onset of the post-glacial warming, genetically diverged lineages of species spread across Europe and where distinct lineages came into contact, inter-lineage admixture led to increased levels of diversity [5–8]. At the leading edges of population expansion, especially in cases where expansion took place from a single refugium, repeated founder events caused decreased levels of diversity, and population fluctuations and demographic stochasticity resulted in increased genetic differentiation among populations [9–11].

For trees, it has been predicted that the leading edge effect during colonization may be less pronounced because of their prolonged juvenile phase. Many migrants have time to reach the newly establishing populations by long-distance dispersal before the first colonizers reach a reproductive age and genetic diversity may thus be maintained [12, 13]. Trees in general are characterized by high genetic diversity within and low genetic differentiation among populations [14] and several studies have shown that genetic diversity in tree species is maintained across large ranges following colonization, even in the absence of mixing between different genetic lineages [9, 15–17]. The longevity of trees, coupled with their high reproduction capacity and ability for long distance dispersal and high levels of pollen mediated gene flow, may thus counteract the effects of colonization [18–20], but this may vary across species and landscapes [16, 17, 21, 22].

Most prior studies on postglacial colonization dynamics of temperate forest tree taxa focused on the southern and central range parts of species because those are the regions from which species expanded in the postglacial and where they are mainly distributed today [7, 9, 23–25]. The northern range margins have received less attention even if they may be particularly suitable to study the genetic processes following postglacial colonization as they have been little affected by northern refugia, c.f. [2]. This contrasts with several boreal tree species whose northern ranges seem to have been more affected by northern refugia [26–28].

Common ash (*Fraxinus excelsior* L., Oleaceae) is a temperate tree species with a wide distribution across Europe. Its distribution ranges from the Mediterranean and the Caspian Sea in the South to mid-Norway (63.5° N) in the North, and from the Atlantic coast in the West into continental Russia in the East [29]. The northernmost distribution of common ash is along the coast of Norway, where it grows in small and fragmented populations [30]. Common ash has been the subject of several genetic studies on range-wide (e.g. [23, 31]), regional (e.g. [32, 33, 34]) and local scales (e.g. [35, 36–38]). From the northern range, there is only one study of four populations from the Finnish archipelago addressing population genetic divergence relative to distance among the island populations [39]. In this study, no reference populations from other parts of the distribution range are included.

Common ash displays genetically distinct chloroplast (plastid) DNA lineages, which are maternally inherited [40], and reflect different postglacial re-colonization routes from the southern European refugia located in Iberia, Italy, the eastern Alps and the Balkans [41]. Based on nuclear microsatellite variation, common ash showed weak genetic differentiation across western Europe owing to pollen-mediated homogenizing gene flow. In south-eastern and eastern Europe however, the nuclear microsatellites indicated regional gene pool structures, probably due to several proximate refugia in the region, substructures that remained undetected with plastid DNA [23]. The elevated levels of genetic differentiation found in common ash populations in southern Sweden [23], southern Finland [39] and northern England [33] have been hypothesized to represent northern remnants of late glacial refugia [23, 33], but no firm evidence exists for such northern refugia.

The aim of this study was to investigate the population history and the genetic structure of northern European common ash populations in relation to central European populations. We collected new population samples from the northern range and analysed them together with reference samples from the central European range available from previously published genetic studies [23, 41].We first placed the northern populations into a phylogeographical framework. Then, we tested if northern range margin populations of common ash from Norway exhibited lower genetic diversity, greater genetic divergence and higher rates of inbreeding than populations in the central European range, as would be expected according to the model of leading edge colonization. Common ash is now threatened across its entire distribution range due to ash dieback [42], a disease caused by the invasive ascomycete *Hymenoscyphus fraxineus* (anamorph *Chalara fraxinea*) presumably originating from Asia [43–47]. The fate of common ash populations may depend on the presence of genetic variation in disease tolerance which may be limited in small and isolated populations due to low genetic diversity (c.f. [48]). Thus an additional aim of our study was to generate baseline information for management and conservation of the northern common ash populations.

## Materials and Methods

### Sampling and genetic data collection

A total of 42 common ash population samples (1099 trees) were analysed covering 16 European countries throughout the species’ distribution range (S1 Table, S1 Fig). The northern range was covered by 17 newly collected Norwegian populations (570 trees; on average 34 individuals per population), 16 of these representing nature reserves regarded as autochthonous forests that have not been influenced by human mediated gene flow. To our knowledge import of ash has been limited to small quantities of plants for use in parks and gardens. Although common ash was commercially important during the days of sailing ships [49], it has never had a significant role in modern forestry in Norway [50].

Due to ash dieback, common ash in Norway was in 2010 considered near threatened and in 2015 vulnerable (Norwegian Red List for Species [51]). Permission to sample common ash and to do fieldwork in the nature reserves were obtained from the County Governors of Hedmark, Buskerud, Akershus, Vestfold, Østfold, Telemark, Aust-Agder, Vest-Agder, Rogaland, Hordaland, Sogn og Fjordane, Møre og Romsdal and Nord-Trøndelag nature management authorities. Addresses are available from https://www.fylkesmannen.no. According to the Norwegian Outdoor Recreation Act, everyone can freely roam and pick flowers, berries, nuts and fungi in the forests. As we picked five leaves from the trees, no additional permission from landowners was necessary.

To enable direct comparison of the genetic constitution of populations in the northern range with southern and central European populations, a set of 25 populations (529 trees) from other parts of Europe was included in the study (S1 Table). Seven of these populations were newly collected for this study, including resampling of the Lithuanian population Kaišiadorys. For the remaining 18 populations, DNA samples were available from previous studies [23, 41].

For the newly sampled populations, DNA was extracted from dried leaf material using the DNeasy 96 Plant Kit (Qiagen) following the manufacturer’s instructions. We assayed four chloroplast DNA microsatellites (cpSSRs; ccmp3, ccmp6, ccmp7 and ccmp10) developed by Weising & Gardner [52] and six nuclear DNA microsatellites (nSSRs); Femsatl4, Femsatl8, Femsatl11, Femsatl16, Femsatl19 originally developed by Lefort *et al*. [53] and one (M2-30) developed by Brachet *et al*. [54]. We used the primers and PCR multiplexing conditions as described in Sutherland *et al*. [33]. All samples (42 populations; 1099 trees) were subjected to nuclear microsatellite analysis. All the newly collected populations (24 populations; 584 trees) and a subset of the DNA samples available from previous studies (18 populations; 246 trees) were subjected to chloroplast microsatellite analysis (S1 Table).

Amplified fragments were separated on an ABI3130 automatic sequencer (Applied Biosystems), fragment sizes and genotypes were determined using GeneMapper Software v. 4.1 (Applied Biosystems by Life Technologies). The coding of the plastid haplotypes followed Heuertz *et al*. [41] and Sutherland *et al*. [33] (Table 1).

**Table 1:** Characteristics of the plastid DNA haplotypes detected for *Fraxinus excelsior* in this study using four chloroplast microsatellite regions. The haplotype definitions follow Heuertz *et al*. [41] and Sutherland *et al*. [33], except for the novel haplotypes H17, H18 and H19. Allele sizes for each of the loci (ccmp3, ccmp6, ccmp7 and ccmp10) are given in base pairs.

Haplotype	ccmp3	ccmp6	ccmp7	ccmp10	Frequency
H01	97	97	118	103	650
H02	97	99	117	104	41
H03	97	99	117	103	26
H04	97	98	118	104	44
H05	97	98	117	103	9
H06	97	97	117	103	22
H07	97	97	118	104	25
H12	96	97	118	103	7
H15	98	99	118	104	1
H17	97	97	117	104	1
H18	98	99	117	104	2
H19	97	97	118	107	2

### Data analysis

#### Genetic diversity and differentiation

Linkage disequilibrium was tested in GENEPOP v. 4.2.2 [55] for all pairs of nSSRs in each population using the log likelihood ratio statistical test. Deviation from Hardy-Weinberg (HW) genotypic proportions was tested using the exact test [56] based on Markov chain iterations in GENEPOP. The extent of deviation from HW proportions was evaluated based on *F*_IS_ estimates (inbreeding coefficient) across loci for each population calculated in *SPAGeDi* v. 1.4 [57]. Critical significance levels of multiple tests were corrected by sequential Bonferroni correction [58]. The presence and frequency of null alleles for each locus and population were estimated following the expectation maximum (EM) algorithm of [59] using FREENA [60]. As some of the loci showed presence of null alleles, we calculated also *F*_IS_ using the Bayesian procedure implemented in INEST v. 2.0 [61], which has been shown to be robust to the presence of null alleles [62]. Statistical significance of inbreeding was assessed by comparing the full model with the random mating model (i.e. when *F*_IS_ is 0) using the Bayesian procedure based on the Deviance Information Criterion (DIC) implemented in INEST.

Genetic diversity was measured for each locus and population as allelic richness (*A*r), observed and expected gene diversity (*H*_O_ and *H*_E,_ respectively) [63] using FSTAT v. 2.9.3.2 [64]. Private allelic richness was calculated in HP-Rare v. 1.1 using rarefaction [65]. Allelic richness was calculated for a sample size of 13 diploids excluding four populations with lower sample size (S1 Table).

Population differentiation was calculated in ARLEQUIN v. 3.5.1.2. [66] based on allele identity and allele size by *F*_ST_ [67] and *R*_ST_, respectively. The significance of genetic differentiation among pairs of populations was tested by 1000 permutations of genotypes among samples. We also calculated standardized measures of population differentiation, Hedrick’s *G*’_ST_ and Jost’s *D*, correcting for differences in diversity and heterozygosity using 1000 bootstrap replicates in SMOGD [68]. We checked the potential effect of null alleles on genetic differentiation by calculating *F*_ST_ values using the excluding null allele (ENA) method by Chapuis and Estoup [60] in FREENA.

#### Population genetic structure

Population genetic structure was analysed employing both spatially informed and non-spatial Bayesian clustering approaches in TESS v. 2.3 [69] and STRUCTURE v. 2.3.4 [70], respectively. For TESS, we first generated individual spatial coordinates and ran TESS without admixture varying the number of clusters (*K*) from 1 to 30, finding the highest possible maximum number of clusters for *K* = 15. Then we used the CAR (Conditional Auto-Regressive) model as described in [71], assuming that the individual genotypes arise from the admixture of *K* unobserved parental populations. We used the default parameters, with a burn-in of 10 000 and run length of 20 000 iterations, for *K* = 1 to *K* = 15 with ten replicates per *K* value. To determine the *K* with the best fit to the genetic data, average Deviation Information Criterion (DIC) values were plotted against *K*. For the STRUCTURE analysis we used the admixture model with correlated allele frequencies, both with and without using location as prior information (LOCPRIOR). Burn-in was set to 500 000 and run length was 500 000 iterations for *K* = 1 to *K* = 10 with ten replicates per *K* value. We used STRUCTURE HARVESTER Web v. 0.6.94 [72] to assess and visualize the likelihood values across the different values of *K*. Similarity coefficients across runs and the average matrices of individual membership proportions were estimated using CLUMP v. 1.1.2 [73]. Clusters were displayed using DISTRUCT v. 1.1 [74]. In addition, the genetic structure was explored with Principal Coordinate Analysis (PCoA) based on the covariance matrix of genetic distances with data standardization as implemented in the program GenAlex v. 6.5. [75, 76]. Compared to the Bayesian clustering approaches this method does not rely on assumptions of HWE or linkage equilibrium.

#### Genetic diversity trends

To identify trends in genetic diversity during colonization, we calculated the correlations among the following parameters: *H*_E_, *A*r, *F*_IS_INEST, latitude and longitude. The correlations were calculated for three different groups of populations: i) all populations representing the entire distribution range of common ash, ii) populations where ancestry was larger than 0.6 in TESS group 2, consisting of most Northern and Eastern European populations representing the putative migration route of common ash from south-eastern Europe to Norway (see results), and iii) Norwegian populations representing the northern range margins. Prior to grouping, we excluded four populations with a sample size < 6 (S1 Table). Correlations (Pearsons’ r) and linear regression coefficients were computed in R v. 3.1.1 [77].

To test whether the genetic differentiation followed an isolation by distance (IBD) model, the significance of the correlation between *F*_ST_/(1-*F*_ST_) and the natural logarithm of geographical distances was tested with a Mantel test [78] using ARLEQUIN with 10 000 permutations. IBD was calculated for the same three groups as above. We tested whether the populations departed from mutation-drift equilibrium using BOTTLENECK v. 1.2.02 where we used the Wilcoxon test to test for heterozygosity excess as this is considered the most powerful and robust test with a limited set of genetic markers [79].

## Results

### Chloroplast haplotypes

**Fig 1:**
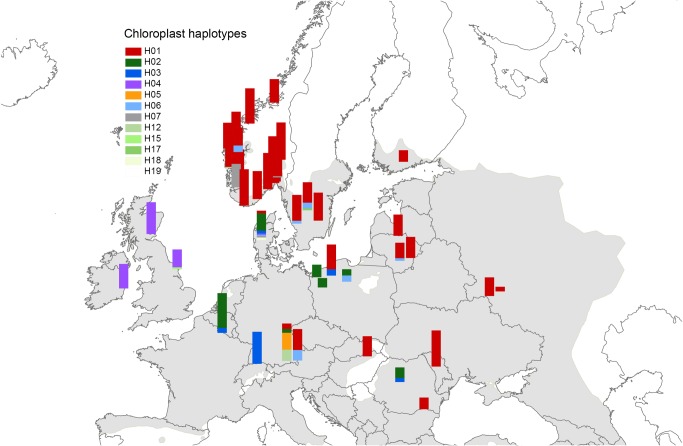
Distribution of chloroplast haplotypes in 42 common ash (*Fraxinus excelsior*) population samples. Coding of the haplotypes follows Heuertz *et al*. [41] and Sutherland *et al*. [33], except for the novel haplotypes H17, H18 and H19. The definitions of the haplotypes are given in Table 1. The shaded gray areas represent the natural distribution range of common ash [29].

In total, 12 plastid haplotypes were detected (Fig 1, Table 1, S1 Table). Haplotype H01, common in eastern and south-eastern Europe [41], was the dominant haplotype in the Norwegian populations. In addition to H01, we identified also haplotypes H06, H07 and H12 in the Norwegian populations with H07 occurring at a high frequency in the south-western population Målandsdalen (8Mal). Three new and rare haplotypes (H17, H18, H19) were identified in single populations in Sweden, Denmark and Norway, respectively (S1 Table); the definition of these low-frequency haplotypes is given in Table 1.

### Nuclear microsatellites

#### Genetic diversity and differentiation

Significant linkage disequilibrium between pairs of loci was detected in 36 of 631 tests (p ≤0.05) after sequential Bonferroni correction. All loci were considered to be genetically independent for further analysis. The single locus tests for HW genotypic proportions within populations showed significant departure in 68 out of 252 cases after Bonferroni correction (p ≤0.05), the significant values corresponded mainly to loci Femsatl8 and Femsatl16. Significant multilocus deviation from HW proportions (*F*_IS_) was observed in 28 populations after sequential Bonferroni corrections (S1 Table; p ≤ 0.05). Taking null alleles into account, the inbreeding coefficient (*F*_IS_INEST) remained significantly different from zero in only nine populations (S1 Table), indicating that the deficiency of observed heterozygosity is mostly due to the presence of null alleles. Assuming HW, the estimated null allele frequencies over populations were highest for the loci Femsatl8 (0.169) and Femsatl16 (0.145), while at the remaining four loci these frequencies were low (<0.02; Table 2). The *F*_IS_INEST values per population were generally low and ranged between 0.010 and 0.077 (S1 Table).

**Table 2:** Basic characteristics of the six nuclear microsatellite loci in *Fraxinus excelsior* used in this study. *A*r, allelic richness, calculated based on 13 diploid individuals; *H*_O_, observed heterozygosity; *H*_E_, expected heterozygosity; *F*_IS_, inbreeding coefficient (the coefficients denoted with asterisk (*) are significantly greater than zero); *F*_ST,_ coefficient of genetic differentiation among populations [63]; and *F*_ST_ENA, coefficient of genetic differentiation calculated using ENA correction [56].

Locus	N alleles	Allele size range	*A*r	*H*_O_	*H*_E_	*F*_IS_	*F*_ST_	*F*_ST_ENA	Null allele frequency
Femsatl4	52	152–266	12.47	0.806	0.879	0.082*	0.049	0.049	0.018
Femsatl8	39	128–196	14.95	0.610	0.944	0.330*	0.043	0.041	0.169
Femsatl11	34	174–242	10.47	0.850	0.889	0.053*	0.061	0.061	0.007
Femsatl16	18	174–208	5.26	0.349	0.641	0.507*	0.094	0.077	0.145
Femsatl19	42	134–225	13.29	0.874	0.931	0.059*	0.054	0.053	0.009
M2-30	52	190–296	17.37	0.890	0.964	0.075*	0.045	0.044	0.011
Mean	40	-	12.35	0.730	0.871	0.168*	0.055	0.053	-

Basic diversity and differentiation characteristics for each of the microsatellite loci are given in Table 2. Population diversity estimates over loci are given in S1 Table. Within populations, the nuclear genetic variation was generally high with gene diversity (*H*_E_) of 0.686–0.920 and allelic richness (*A*r) of 5.417–12.567 (based on 13 diploid individuals; S1 Table).Thirty-three private alleles were detected, all of which corresponded to extremes of the allele size range. Private allelic richness ranged from 0–0.520, with a mean value of 0.130 and a standard deviation (SD) of 0.120. The private alleles showed no apparent geographic structure, but several Norwegian populations (15Hind, 14Mjo, 10Kly, Fj and No; S1 Table) showed slightly elevated levels of private allelic richness. The overall population genetic differentiation was low with *F*_ST_ = 0.055 (SD = 0.005) and *R*_ST_ = 0.052 (SD = 0.015), both values significantly greater than zero (Table 2). After ENA correction, the overall *F*_ST_ didn’t change (*F*_ST_ENA = 0.053; Table 2). Hedrick’s *G*’_ST_ and Jost’s *D* showed higher genetic differentiation, 0.396 and 0.309, respectively. Population pairwise *F*_ST_ values ranged from 0–0.190 (S2 Table). The highest pairwise *F*_ST_ values were obtained for pairs including some Norwegian populations: the northernmost population 15Hind (average pairwise *F*_ST_ = 0.135) and three populations on the Norwegian northwest coast; 14Mjo (average pairwise *F*_ST_ = 0.091), 12Ron (average pairwise *F*_ST_ = 0.083) and 13Asa (average pairwise *F*_ST_ = 0.063).

#### Genetic structure

**Fig 2:**
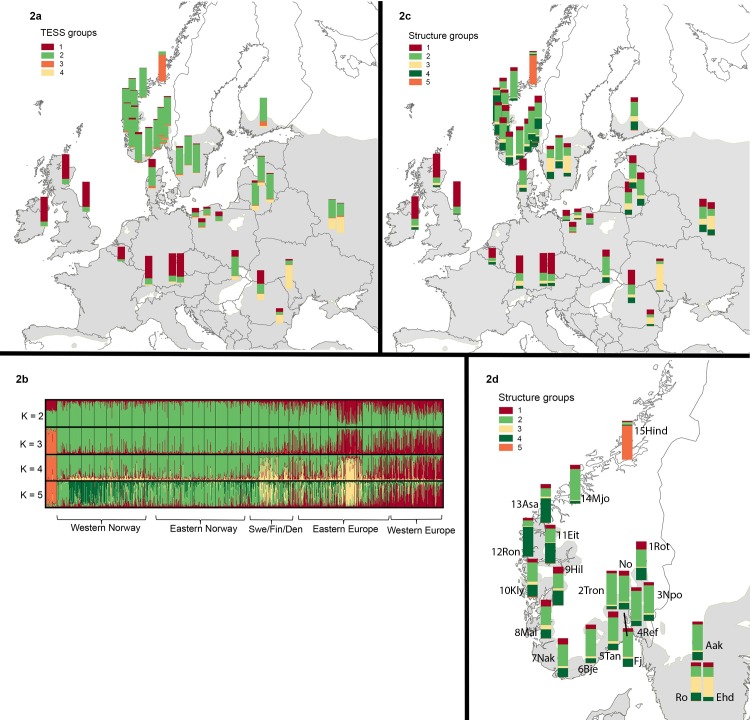
Distribution of TESS (2a) and STRUCTURE (2c, d) groups in common ash (*Fraxinus excelsior*) populations. 2b shows bar plots of clustering based on STRUCTURE for *K* = 2–5. The shaded gray areas represent the natural distribution range of common ash [29].

Both TESS and STRUCTURE identified genetic subdivision along longitudinal and latitudinal gradients (Fig 2). For TESS, the lowest DIC value and visual inspection of the decay of DIC suggested that the main structure of the data was captured at *K* = 4 (Fig 2A). One TESS group (group 1) was found across western and central Europe and included parts of a population in Romania. The second TESS group (group 2) included mainly northern European and Baltic populations along with parts of Hungarian, Romanian and Russian populations. The third TESS group (group 3) consisted mainly of the northernmost Norwegian population 15Hind in Trøndelag. The fourth TESS group (group 4) was found at highest frequencies in the populations in Moldova, Bulgaria and Russia (Fig 2A).

In STRUCTURE, the posterior likelihood (LnPD) increased progressively as *K* increased, reaching a plateau at *K* = 6. As *K* = 6 gave very little additional information compared to *K* = 5 and the main structure of the data was captured at *K* = 5, we present the geographical distribution of *K* = 5 (Fig 2B). Several STRUCTURE groups were represented in populations from south-eastern, eastern and northern Europe (Fig 2C). The proportions assigned to the different groups were however asymmetrical suggesting that there is a genuine population structure. The STRUCTURE grouping was similar to the TESS grouping with the exception of the northern range where several of the north-western Norwegian populations contained a high percentage of a STRUCTURE group 4 (Fig 2D, S1 Table). As in TESS, the northernmost population from Trøndelag (15Hind) constituted a separate group (STRUCTURE group 5).

**Fig 3:**
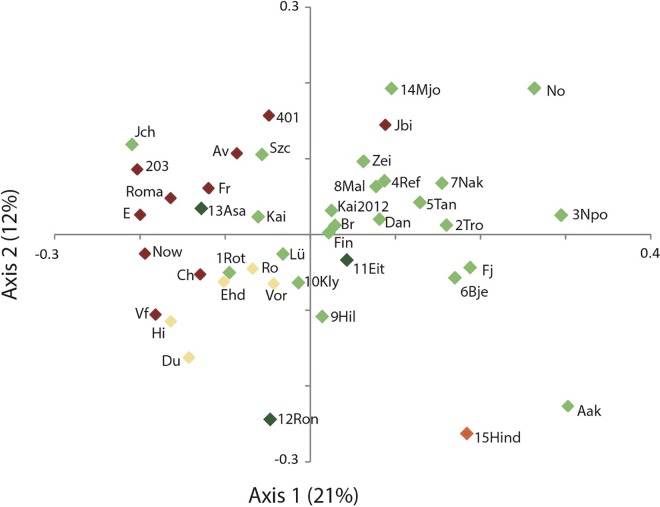
Principal Coordinates Analysis (PCoA) plot of the first and the second principal coordinates based on the genetic composition at six nuclear microsatellites of 42 population samples of common ash (*Fraxinus excelsior*). For population names see S1 Table and S1 Fig. Each population is given the color of the STRUCTURE group (Fig 2C) in which it had the highest proportion of membership (S1 Table).

The two first axes of the PCoA explained 21% and 12% of the variation, respectively (Fig 3). The distribution of the populations in the plot largely reflected the STRUCTURE groups with diffuse clusters of western, eastern and northern populations. The Norwegian populations were spread out along axis 1, except the northernmost populations (12Ron, 13Asa, 14Mjo and 15Hind), which were scattered across the PCoA plot (Fig 3), a pattern coherent with their high pairwise *F*_ST_ values.

#### Patterns of genetic diversity

**Fig 4:**
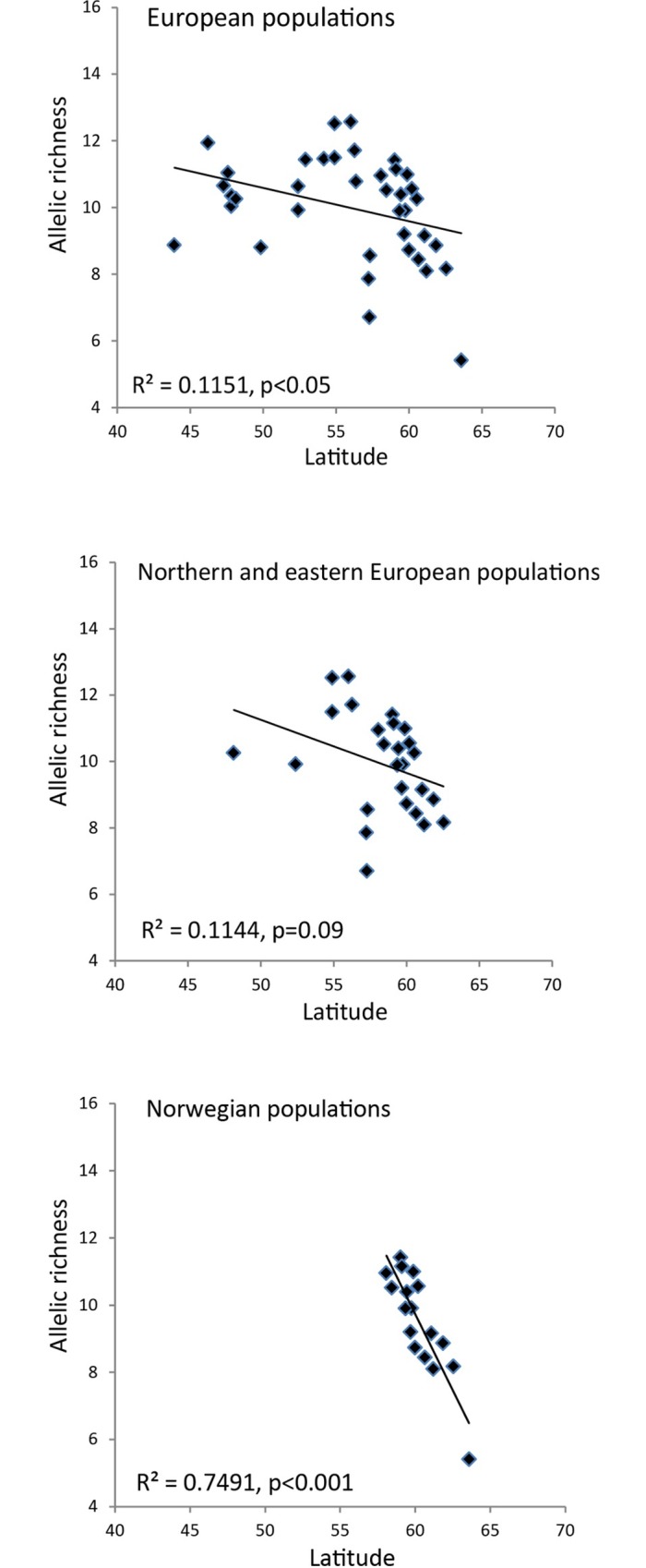
**Linear regression between allelic richness and latitude for common ash (*Fraxinus excelsior*) populations a) across Europe overall, b) across northern and eastern European populations putatively constituting colonization from south-eastern Europe northwards (TESS group 2) and c) across Norwegian populations.** Allelic richness is calculated based on a random set of 13 diploid individuals per population.

Within Norway we detected a strong and significant (p < 0.001) negative correlation between *A*r and latitude as well as between *H*_E_ and latitude (Table 3, Fig 4). This is in contrast to Europe overall where *A*r and latitude were weakly negatively correlated (although significant at p ≤ 0.05). Within TESS group 2, no significant correlations were observed between *A*r, *H*_E_ and latitude. In all groups, *A*r and *H*_E_ were strongly correlated. No significant correlations were found between genetic diversity and longitude, but *F*_IS_INEST was positively correlated with longitude both in Europe overall and in TESS group 2, but not in Norway alone (Table 3). The patterns of genetic diversity were similar across all loci (results for single loci are not shown). Patterns of diversity within the other gene pools were found to be similar to those reported by Heuertz *et al*. [23] and are thus not further analysed or discussed here. The genotype data for the nuclear microsatellite loci are available in S4 Table.

**Table 3:** Pearson’s correlation coefficients (r) between allelic richness (*A*r), gene diversity (*H*_E_), inbreeding coefficient calculated with INEST (*F*_IS_INEST), latitude and longitude calculated for populations within: (i) Europe overall, (ii) TESS group 2 (constituting populations from northern and eastern Europe, see S1 Table), and (iii) Norway.

Group	Variable	*A*r	*H*_E_	*F*_IS_INEST
Europe overall	Latitude	-0.339*	-0.285	-0.222
(n = 38)	Longitude	0.110	0.055	0.397*
	*A*r		0.899***	0.088
	*H*_E_			0.122
TESS group 2	Latitude	-0.338	-0.336	-0.258
(n = 26)	Longitude	0.258	0.282	0.688***
	*A*r		0.916***	0.104
	*H*_E_			0.203
Norway	Latitude	-0.865***	-0.782***	0.241
(n = 17)	Longitude	-0.049	-0.071	0.343
	*A*r		0.931***	-0.379
	*H*_E_			-0.326

* and *** denote significant correlations at the 5 and 0.1% levels, respectively.

#### Isolation by distance and bottleneck tests

There was no IBD neither across Europe overall (R^2^ = 0.00006, p = 0.460), nor within TESS group 2 (R^2^ = 0.0034, p = 0.306). Within Norway, there was a weak though significant IBD pattern (R^2^ = 0.068, p = 0.020). No consistent evidence for bottlenecks (i.e. heterozygote excess under all three mutation models) was found in any of the populations. Fifteen populations showed significant heterozygote excess in the Wilcoxon test, either under the infinite allele model or the two phase model (S3 Table). In thirteen of these populations however, a significant heterozygote deficiency was detected under the stepwise mutation model. The two populations left with a sign of bottleneck were population La Romagne in France, and population 401 in Great Britain (S3 Table).

## Discussion

### Phylogeographic history of the common ash populations in northern Europe

All common ash populations from northern Europe showed the plastid DNA haplotype H01, which is characteristic to eastern and south-eastern European populations [41]. The gene pool structure of nuclear microsatellite data additionally linked the northern range populations to eastern and south-eastern European populations. The unusual plastid DNA haplotypes H06 and H07 detected in the west coast populations in Norway may originate from long distance dispersal, but they can also reflect human-mediated gene flow or homoplasious mutations. Haplotype H07 has been observed earlier in populations from Ireland and Romania, between which homoplasy was demonstrated based on cpSSR vs. PCR-RFLP allelic associations [41]. Both the plastid and the nuclear genetic data thus suggest that the northern range of common ash was colonized via a single migration route that originated in eastern or south-eastern Europe with little influence from other southern or western European refugia.

In south-eastern Europe, pollen diagrams indicate a late glacial presence of common ash in the Balkan peninsula [80–82]. Pollen diagrams from the northern Alps and the Carpathians document rapid early Holocene population expansions suggesting that the full glacial distribution of common ash was large and may have included regions as far north as the Carpathian Basin [83]. The previously published nuclear microsatellite data suggested expansion from refugia located in the western Balkan Peninsula and north-eastern Europe with admixture in Slovakia and Hungary [23]. Expansion northwards may have taken place out of this region. Based on the pattern of cpDNA presented in Heuertz *et al*. [41], migration northwards took place following two broad parallel migration routes. These migration routes seem to have met in north-western Poland at the border to Germany where both haplotype H01 and H02 are found (Fig 1), see also [41]. A contact zone in northern Poland can also explain the genetic patterns in the Polish populations revealed by both TESS and STRUCTURE (Fig 2). When using 0.5% of terrestrial pollen as an indication of regional presence of *Fraxinus*, it can be deduced that the taxon was present in Poland and the Baltic States around 9 cal kyr BP (pollen threshold 2–4%), and in southern Sweden 8 cal kyr. BP (0.5–1% pollen threshold) [81, 82]. In this period a mainland connection existed between southern Sweden and northern Germany/Denmark following the Ancylus-regression of the Baltic Sea [84]. This mainland connection may have been important for the dispersal of *Fraxinus* seeds along with other species into Scandinavia. As only haplotype H01 dispersed to Sweden and Norway, it may indicate that H02 and the other haplotypes found in Denmark arrived after the connection to Sweden was broken. Denmark is thus not the source of colonization to Sweden and Norway, which is in contrast to what has been found in e.g. oak [85]. Two of the Swedish population have high frequencies of STRUCTURE group 3, a group found in Moldavia and Russia, and there may have been additional migration routes from the south or from the east at different times. The Swedish populations have in addition low allelic richness, which may suggest founder events. Denser pollen records [81, 82] and more genetic data would however be necessary to trace the different migration routes.

Our nuclear microsatellite analysis suggests that the northernmost population in Norway, Trøndelag (15Hind), constitutes a separate genetic group with very low levels of genetic diversity. Its genetic peculiarity is probably due to a shift in allele frequencies, either because of a founder event following recent long-distance colonization, or owing to drift following long-term persistence of a small ash population in the region. The slightly elevated private allelic richness in the population may also have contributed to its genetic partition, although it is still lower than the mean value plus standard deviation (0.130; SD 0.120) for all the populations. Interestingly, *Fraxinus* pollen (0.5–1%) was detected in the Trøndelag region in the early Holocene (at 7.5 and 6.0 cal kyr BP [81, 82]). Pollen records from the central Scandes Mountains, a region close to Trøndelag, suggested an early Holocene forest with *Corylus avellana*, *Alnus glutinosa* and *Ulmus glabra* as common constituents, along with rare occurrences of *Tilia cordata* [86]. *Fraxinus excelsior* could possibly have been one of the rare constituents of such an early Holocene forest in the region. Upon climate cooling during the late Holocene these forests mostly disappeared, and the current isolation of the 15Hind population is likely promoting its divergence. They population may expand as if flowers and sets seed (personal observation).

### Contrasting postglacial colonization dynamics across the range

In the northern range of common ash in Norway, we detected a steep latitudinal decrease in both allelic richness and expected heterozygosity. Increased genetic differentiation among populations was evident from the PCoA plot (e.g., populations 14Mjo, No, 13Asa, 12Ron, 15Hind; Fig 3), high pairwise *F*_ST_ values (S2 Table), and from Bayesian clustering analyses (Fig 2). Such a genetic pattern with decreasing within population diversity and increasing population differentiation is consistent with a leading edge colonization model [10]. In Norway, common ash is mainly restricted to a narrow nemoral forest belt along the coast, etched by fjords, valleys and high mountain ridges. Colonization of this coastal range was probably one-dimensional, and occurred likely through repeated establishments of small, isolated populations from the leading colonizing front. Moreover, long-distance dispersal events [87] were probably important to overcome the physiographical barriers, but not frequent enough to counteract the loss of genetic diversity through drift related to the founder effect. In common ash, pollen dispersal has been estimated to be much more pronounced than seed dispersal [36], but the fragmented landscape along the Norwegian coast probably significantly reduced the pollen exchangeability. The prevailing wind direction along the Norwegian coast in late spring/summer time is additionally from north to south, so pollen flow from south to north are probably not frequent. In common ash, seeds stay on the tree until they are wind-dispersed during wintertime. During wintertime, the winds are mainly from south to north, possibly facilitating seed dispersal northwards.

The complex pattern of population genetic structure and the weak IBD within Norway may also be related to historical population size changes. Pollen profiles indicate that the abundance of common ash in Scandinavia has declined over the last 4000 years [81], possibly reinforcing fragmentation and enhancing genetic drift, although no signal of bottleneck were observed in the northern range populations. In common ash, fruiting phenology varies with both latitude and temperature [88], and northern climates may constrain gene flow via reduced pollen and seed production.

The genetic pattern in the northern range contrasts with data from continental Europe where genetic diversity decreases only weakly with increasing latitude. In central and eastern Europe, pollen records suggested a very rapid re-colonization of *F*. *excelsior* [83]. Colonization of the western and central European range probably involved repeated long-distance dispersal events during population establishment and admixture of distinct lineages, these leading to high genetic diversity and weak genetic differentiation among populations [23]. In addition, increased pollen exchange due to larger population sizes [38] probably maintained diversity and antagonised genetic differentiation more efficiently in the central and southern European range [23] than in the northern range. Our data from the northern range in Norway, also contrast with the data from Ireland [34] and Britain [33] where common ash basically constitutes one large gene pool, with only little differences in genetic diversity among populations.

Based on the central-marginal hypothesis, loss of genetic diversity and greater genetic differentiation in the range margins may be a consequence of smaller effective population size and greater geographical isolation relative to more abundant central populations (e.g. [89, 90–93]). When Eckert *et al*. [94] sought to quantify the support for a central-peripheral hypothesis they found that genetic diversity declined towards the range margins in 64.3% of the studies. In most cases however, the difference in genetic diversity between central and peripheral populations was small, and almost no studies incorporated a phylogeographic framework to evaluate the historical influences on the contemporary genetic pattern, but see e.g. [95, 96]. In our study, the loss of genetic diversity and increase in genetic divergence in common ash’s northern range is pronounced. Set into a phylogeographic context, postglacial history seems to remain the central determinant of the genetic structure of common ash in the northern range despite the constraints associated with marginality.

### Low inbreeding

Based on the Bayesian calculations in INEST [61], there were only two populations in Norway with inbreeding coefficients significantly larger than zero (*F*_IS_INEST). Although some bi-parental inbreeding may be expected [35], the estimates based on INEST suggest that natural common ash populations are characterised by very low inbreeding, even at the northern range margins. Common ash displays a complex, polygamous sexual system where individual trees can be phenotypically classified from pure males to pure females with a range of hermaphrodite intermediates. Controlled pollination showed that pollen from male trees have an advantage over pollen from hermaphrodite trees [97], and common ash has been confirmed to be predominantly outcrossing in other studies (e.g. [37]). We observed *F*_IS_INEST values significantly larger than zero in some of the eastern populations (e.g. from Finland (Fin) and Lithuania (Kai2012); S1 Table), explaining the correlation between *F*_IS_INEST and longitude within TESS group 2. In Finland, common ash has a very scattered and restricted distribution and microsatellite analysis of four Finnish island populations suggested an almost total interruption of gene flow among populations [39], probably enhancing breeding among relatives. In the Lithuanian Kaišiadorys population sampled in 2012, the forest was heavily affected by ash dieback and the samples were mainly collected from the younger generation of surviving trees, possibly representing offspring between related surviving trees. In other studies, higher *F*_IS_ estimates have been observed, e.g. in British [33], French [98] and Italian [32] samples, possibly due to null alleles or due to the Wahlund effect. In the Irish study where more loci were used, much lower *F*_IS_ values were observed [34]. The differences in *F*_IS_ values among studies are probably partly due to loci and the methods used for calculating *F*_IS_ as well as actual differences in inbreeding.

### Management and conservation implications for northern European common ash populations

Ash dieback epidemic is currently threatening common ash at continental scale in Europe [42]. A significant loss of common ash stands is observed in many European countries; for example, in Lithuania sanitary clear-fellings due to ash dieback have resulted in a significant shift in species composition towards grey alder and birch [99]. Based on field data available from several countries, McKinney *et al*. [100] estimated that only 1–5% of the trees in native populations of common ash possess notable resistance against the ash dieback pathogen. Based on the idea that allelic diversity is the main source of variation with regard to long-term response to selection [101, 102], the low allelic diversity in the northern common ash populations may jeopardise their adaptive potential if exposed to ash dieback. In addition to being genetically depauperated, the northernmost populations are also small and isolated, which make them particular vulnerable to new selection pressures (c.f. [48]). However, due to their genetic distinctness (we find e.g. strong allele frequency shifts in the northernmost population in Trøndelag) they could constitute a valuable genetic resource that would warrant conservation (c.f. [103]). Given the limited gene flow among populations in the northern range margin, the ash dieback epidemic may eventually cause detrimental inbreeding in these populations [104].

## Conclusions

Trees are generally considered to be relatively resilient to the effect of small population size and increased isolation due to their longevity, high reproductive capacity, and ability for long distance seed dispersal and pollen flow. In common ash, populations in the central distribution range show high genetic diversity and low genetic differentiation, whereas populations in the northern range margins show lower diversity and increased divergence, most probably as a consequence of a leading edge colonization process towards the north. In the northern range in Norway, common ash is mainly restricted to a narrow nemoral forest belt along the coast and colonization of this range was probably one-dimensional, involving repeated establishments of small and isolated populations from the leading colonizing front followed by drift. The northern populations were, however, not affected by inbreeding. Currently, in common ash a strong selection pressure due to the ash dieback epidemic is on-going. The current low genetic diversity in the scattered northern range populations of common ash may compromise their future distribution.

## Supporting Information

S1 Table Location, frequency of chloroplast haplotypes and population genetic estimators at nuclear microsatellite data for 42 *Fraxinus excelsior* populations. (XLSX)

S2 Table Pairwise *F*_ST_ values between 42 population samples of *Fraxinus excelsior* based on six nuclear microsatellite loci. (XLSX)

S3 Table Results from the bottleneck test. The Wilcoxon test was used under the infinite allele model (IAM), two phase model (TPM) and the stepwise mutation model (SMM).
(XLS)

S4 Table Genotypes at six nuclear microsatellite loci for 42 *Fraxinus excelsior* populations. (XLSX)

S5 Table Overview of the nature reserves and the contact persons at the County Governors nature management authorities who issued the permission to collect *Fraxinus excelsior* in the different locations. (XLSX)

S1 Fig Map showing the locations and abbreviated names of the 42 analysed *Fraxinus excelsior* populations. (TIF)
