# Supplementary figures and images for: Genetic Structure in the Northern Range Margins of Common Ash, Fraxinus excelsior L
Source: PLoS One. 2016 Dec 1;11(12):e0167104. doi: 10.1371/journal.pone.0167104 (PMC5132317; doi:10.1371/journal.pone.0167104)

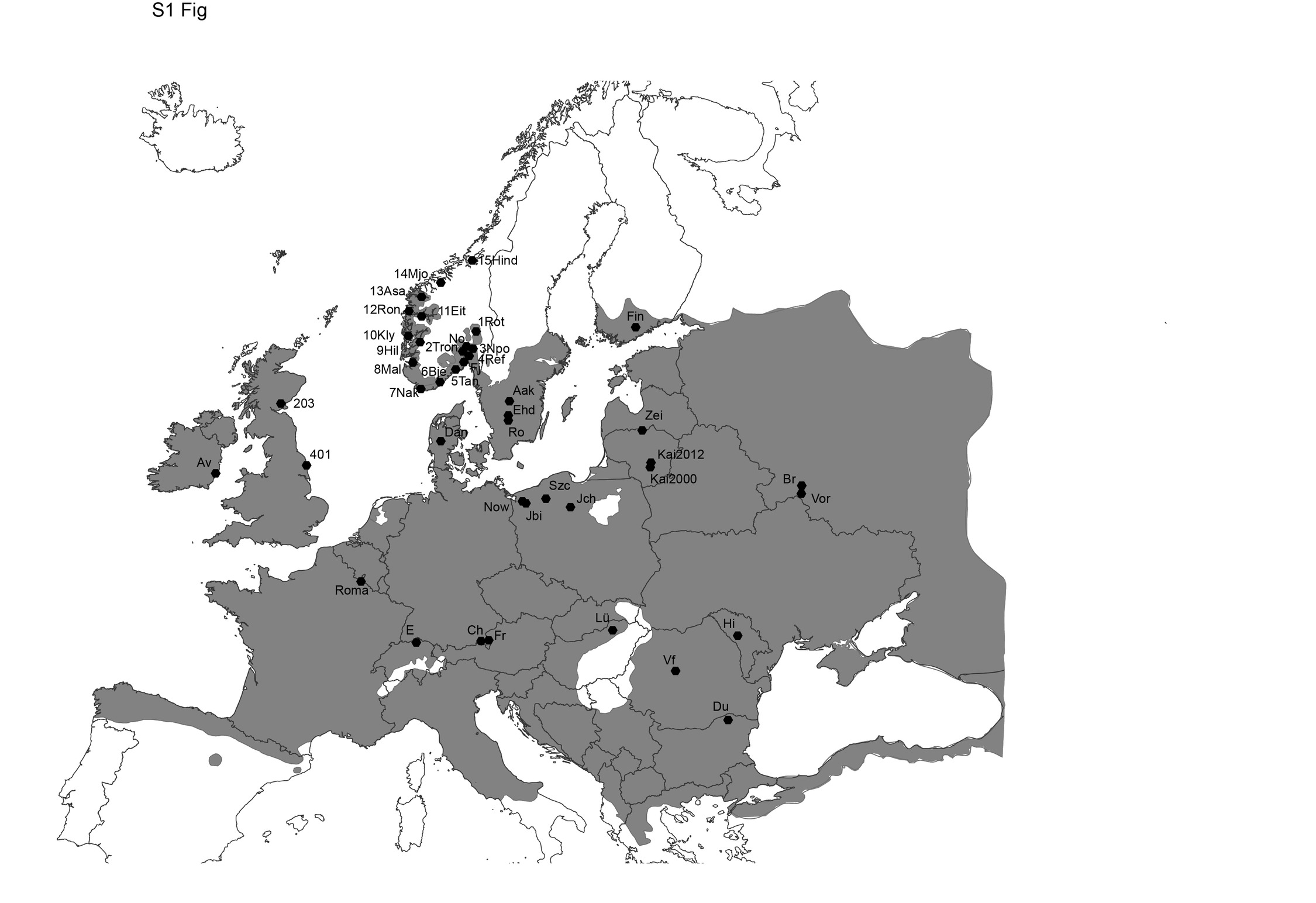

Supplement: S1 Fig — (TIF) [file pone.0167104.s006.tif]
